# Clinical efficacy and safety evaluation of camrelizumab plus lenvatinib in adjuvant therapy after hepatocellular carcinoma surgery

**DOI:** 10.3389/fonc.2023.1174999

**Published:** 2023-12-13

**Authors:** Xudan Wang, Weiwei Cao, Yan Qiu, Hongchen Ji, Juzheng Yuan, Weikang Wu, Fuyuan Liu, Liangyong Feng, Rui Ding, Xiao Li, Kaishan Tao

**Affiliations:** ^1^ Department of Hepatobiliary Surgery, Xijing Hospital, Fourth Military Medical University, Xi’an, Shaanxi, China; ^2^ Department of Clinical Laboratory, Xijing Hospital, Fourth Military Medical University, Xi’an, Shaanxi, China; ^3^ Department of Oncology, Xijing Hospital, Fourth Military Medical University, Xi’an, Shaanxi, China; ^4^ Department of General Surgery, Xijing Hospital, Fourth Military Medical University, Xi’an, Shaanxi, China

**Keywords:** hepatocellular carcinoma, camrelizumab, targeted drugs, adjuvant therapy, survival, adverse events

## Abstract

**Objective:**

To assess the efficacy and safety of camrelizumab plus different targeted drugs in adjuvant therapy after hepatocellular carcinoma (HCC) surgery.

**Patients and methods:**

This retrospective cohort study included HCC patients who, after undergoing failed postoperative adjuvant lenvatinib therapy, received intravenous camrelizumab 200 mg every 3 weeks (C group, *n* = 97), camrelizumab plus oral apatinib 250 mg daily (C+A group, *n* = 125), camrelizumab plus oral lenvatinib 12 mg daily (for bodyweight ≥60 kg)/lenvatinib 8 mg daily (for bodyweight <60 kg) (C+L group, *n* = 120), or camrelizumab plus oral sorafenib 400 mg bi-daily (C+S group, *n* = 114) between October 2020 and October 2021. The outcomes including the objective response rate (ORR) and disease control rate (DCR) were evaluated by RECIST 1.1 and iRECIST. The median progression-free survival (mPFS), median overall survival (mOS), 6-month OS rate, 12-month OS rate, and adverse events were evaluated.

**Results:**

As of 31 May 2022 with last follow-up time, the ORR was 17.2% for the C group, 44.6% for the C+A group, 47.9% for the C+L group, and 36.3% for the C+S group. The DCR was 72.0% for the C group, 81.8% for the C+A group, 85.5% for the C+L group, and 77.9% for the C+S group. The mPFS was 11.0 months (10.1–12.8) for the C group, 14.0 months (12.7–16.5) for the C+A group, 18.0 months (16.9–20.1) for the C+L group, and 12.0 months (9.7–14.4) for the C+S group. The mOS was 13.0 months (11.6–15.3) for the C group, 17.0 months (15.8–19.4) for the C+A group, 19.0 months (17.7–20.2) for the C+L group, and 15.0 months (14.1–17.3) for the C+S group. Grade 3 or 4 treatment-related adverse events occurred in 14 patients (14.4%) for the C group, 10 patients (8.0%) for the C+A group, 5 patients (4.2%) for the C+L group, and 11 patients (9.6%) for the C+S group. The most common adverse events were fatigue and transaminitis.

**Conclusion:**

Camrelizumab combined with lenvatinib as adjuvant therapy showed promising efficacy and manageable safety in HCC patients. It might be a potential adjuvant therapy or second-line treatment for these patients.

## Introduction

Hepatocellular carcinoma (HCC) is the most common malignancy in primary liver cancer, accounting for more than 85% of primary liver cancer, which has no specific clinical symptoms after the onset of the disease, but its development, spread, and metastasis are fast (1). The traditional method of treating liver cancer is surgical resection. However, because of the high invasiveness of malignant tumors, the 5-year survival rate of HCC patients after surgical resection is still low, and the tumor recurrence rate ranged from 50% to 70% (2). This directly leads to its incidence ranking fourth worldwide and its mortality rate ranking third (3). Therefore, exploring more effective adjuvant therapy to prevent HCC recurrence after liver resection and improve the survival benefits of patients with HCC has always occupied an important position in clinical and scientific research.

Programmed cell death protein 1 or ligand 1 (PD-1/L1) inhibitors have shown promising antitumor activity in a variety of tumors. However, due to the complex tumor microenvironment, the efficacy of PD-1/L1 inhibitor monotherapy is not ideal in the treatment of gastrointestinal malignancies. It may be that the presence of a large fibrous stroma around the tumor matrix can hinder T-cell infiltration (4), although an increasing number of studies have shown that the genomic characteristics and tumor microenvironment characteristics of liver cancer may predict benefits from immunotherapy (5). For HCC, the objective response rate (ORR) of PD-1 immunosuppressive monotherapy is approximately 4%–20% (6). The PD-1 inhibitor camrelizumab (SHR-1210) has been proven to block the binding of PD-1 to PD-L1, thereby inhibiting the immune escape of tumor cells. A clinical phase 2 trial of patients with advanced HCC showed that the ORR of camrelizumab was just 11.9% (given every 2 weeks) and 17.6% (given every 3 weeks) (7). A global, randomized, double-blind, placebo-controlled phase 3 trial indicated that the median disease-free survival was significantly increased in the patients who received adjuvant nivolumab treatment compared with the patients who received placebo (22.4 months vs. 11.0 months) in resected esophageal or gastroesophageal junction cancer, but 34% of patients still had grade 3 to 4 adverse reactions, leading to discontinuation of trials in some patients (8). Therefore, immune checkpoint inhibitors have brought novel and promising opportunities, whether as second-line or adjuvant therapy, but they remain less satisfactory due to poor efficacy or serious adverse effects. How to improve their efficacy and reduce their adverse reactions has become the key to tumor treatment or adjuvant therapy.

A variety of signaling factors, such as fibroblast growth factor receptors (FGFRs) and vascular endothelial growth factors (VEGFs), play an important role in the occurrence and development of HCC (9, 10). Preclinical models seem to suggest that drugs targeting VEGF, FGFR, epidermal growth factor receptor (EGFR), and other signaling pathways can improve the tumor microenvironment and reshape the immune response to inhibit tumor progression (11). In the current research on inhibitors of the VEGF pathway, sorafenib, cabozantinib, and sunitinib have all been studied in clinical trials in HCC, but their monotherapy effect is less than satisfactory (ORR, 4%, 4%, and 2.7%) (12–14). FGFR plays a significant role in regulating the proliferation, migration, invasion, and angiogenesis of HCC cells, and tyrosine kinase inhibitors (TKIs) targeting this pathway are currently widely developed. Both apatinib and lenvatinib are multi-target TKIs; the former can inhibit vascular endothelial growth factor receptor (VEGFR)-2 highly selectively (15), and the latter can inhibit VEGFR-1–3, FGFR1–4, platelet-derived growth factor receptor (PDGFR)-α, stem cell factor receptor (KIT), glial cell-derived neurotrophic factor receptor (RET), and other tumor-related targets (16, 17). A study of patients with primary liver cancer showed that the ORR of apatinib was 16%, and median overall survival (OS) was 13 months (18). Phase 2 study in patients with advanced HCC indicated that the ORR of lenvatinib was 37%, and median OS was 18.7 months (19). In addition, lenvatinib also showed good antitumor efficacy in endometrial cancer, kidney cancer, and thyroid cancer (20). Considering the potential synergistic effect of targeted therapy and immune checkpoint inhibitors, targeted drugs combined with camrelizumab may be a potentially effective way to treat various tumors.

Our retrospective study evaluates the efficacy and safety of different targeted drugs (sorafenib, apatinib, and lenvatinib) plus camrelizumab in HCC patients after undergoing postoperative adjuvant lenvatinib therapy failure to choose an optimal treatment strategy, in order to provide a preliminary basis and reference experience for adjuvant therapy of HCC.

## Patients and methods

### Patient selection

This retrospective study collected 2,182 patients with histologically confirmed HCC from the Department of Hepatobiliary Surgery and the Department of Oncology in our hospital between October 2020 and October 2021. Among the 2,182 patients included in this study, there were 1,174 male and 1,008 female patients, with an average age of 55.83 ± 10.26 years. A total of 1,811 patients had a history of smoking, 2,073 patients were HBV-infected, and 982 patients had an AFP level ≥400 ng/mL. Among them, 816 patients underwent the treatment with open partial hepatic resection and postoperative lenvatinib first-line adjuvant therapy failure. After excluding ineligible and lost-to-follow-up patients, the remaining 456 patients were included in the study. Then, these patients received camrelizumab monotherapy or camrelizumab plus the three targeted drugs. We collected the clinical medical records of these patients, including basic information of patients, laboratory test results, and imaging data, and determined adverse reactions, disease progression and time of death through follow-up (deadline: May 2022). This retrospective study was approved by the Ethics Committee of the First Affiliated Hospital of the Air Force Military Medical University (No. KY20172013-1). All patients have informed consent.

The inclusion criteria were as follows:

1) Age ≥ 18 years.
2) HCC diagnosis was based on histological examination or the criteria of the American Association for the Study of Liver Diseases (AASLD) guidelines (21).
3) Clinical data and follow-up records were complete.
4) Child‐Pugh liver function class: A.
5) All patients were treated with open partial hepatectomy, and postoperative underwent adjuvant therapy failure with oral lenvatinib 12 mg daily (for bodyweight ≥60 kg)/lenvatinib 8 mg daily (for bodyweight <60 kg) (Eisai, Co., Ltd., Tokyo, Japan). The inclusion criteria for selecting patients for adjuvant lenvatinib treatment after surgery are as follows: the presence of large enhanced lesions on CT scan, Child–Pugh liver function class A, PS score of 0–1, or incomplete surgical resection or presence of residual lesions. Treatment failure is defined as the detection of intrahepatic recurrence or extrahepatic metastases on CT scan after lenvatinib treatment, along with elevated levels of AFP compared to post-hepatectomy levels, indicating disease progression and recurrence. There were no contraindications to immune and targeted therapy.
6) According to the Response Evaluation Criteria in Solid Tumor 1.1 (RECIST 1.1) (22), and the Immune Response Evaluation Criteria in Solid Tumors (iRECIST) (23), at least one measurable lesion was visible on imaging.
7) As of the time of follow-up, patients received at least two doses of camrelizumab monotherapy or camrelizumab plus the three targeted drugs therapy.

The exclusion criteria were as follows:

1) Concomitant with other malignancies.
2) Severe comorbidities such as liver, kidney, heart, and brain dysfunction or infection.
3) Incomplete clinical data.
4) Efficacy had not been or could not be evaluated (imaging data were lacking during follow-up, follow-up was lost, or patients died before the first efficacy assessment).
5) Patients had a history of severe hypertension that cannot be controlled by conventional medications.
6) Combined with other surgeries, such as cholecystectomy, removal of portal vein tumor thrombus, partial diaphragmatic resection, T-tube drainage, chemotherapy particle implantation, etc.
7) Patients who previously or concurrently received other immunotherapies (such as anti-PD-L1, PD-L2, or CTLA-4 therapy) in the past 6 months.
8) Patients who simultaneously received chemotherapy or topical therapy (including radiofrequency ablation, microwave ablation, or TACE) in the past 6 months.
9) Patients with treatment interruption.

### Methods of open partial hepatectomy

The patient was placed in the supine position under general anesthesia, the hepatic area was appropriately raised, and partial liver resection was performed. An oblique incision or an inverse L-shaped incision under the right costal margin, approximately 15–25 cm in length, was made. Furthermore, we freed the adhesions around the liver, broke the right triangular ligament of the liver, and exposed the lesion. A blocking band was present at the first hepatic hilum, and the location and size of the tumor were determined under direct vision or ultrasound guidance during the operation. The place approximately 2 cm from the edge of the tumor was sutured with No. 7 silk thread as a noose, and the suture closed around. Then, the first hepatic hilum was blocked, and the liver tumor was excised along the inner side of the noose suture and taken out. End face stitching and surgical hemostasis were done. After releasing the first hepatic hilum and observing no clear bleeding point, we further washed the wound, placed the drainage tube, and closed the abdomen.

For deep-seated lesions within the liver, we performed anatomical liver resection or segmental/lobar resection to ensure adequate resection coverage. In instances where the liver’s anatomical position presented challenges, making anatomical resection unfeasible, we employed ultrasound guidance to accurately define the tumor’s boundaries and proceed with an irregular resection approach.

### Treatment protocol

The patients were divided into four groups, including the camrelizumab monotherapy group (C group, *n* = 97), camrelizumab plus apatinib group (C+A group, *n* = 125), camrelizumab plus lenvatinib group (C+L group, *n* = 120), and camrelizumab plus sorafenib group (C+S group, *n* = 114).

1) The patients in the C group received intravenous camrelizumab 200 mg every 3 weeks (Jiangsu Hengrui Pharmaceuticals Co., Ltd., Jiangsu, China).

2) The patients in the C+A group received intravenous camrelizumab 200 mg every 3 weeks plus oral apatinib 850 mg daily (Jiangsu Hengrui Pharmaceuticals Co., Ltd., Jiangsu, China).

3) The patients in the C+L group received the intravenous camrelizumab 200 mg every 3 weeks plus oral lenvatinib 12 mg daily (for bodyweight ≥60 kg)/lenvatinib 8 mg daily (for bodyweight <60 kg).

4) The patients in the C+S group received the intravenous camrelizumab 200 mg every 3 weeks plus oral sorafenib 400 mg bi-daily (Bayer Pharma AG, Leverkusen, Germany).

Patients received immunotherapy for at least 2 cycles and targeted therapy for at least 42 days. All patients were routinely checked for blood routine, liver and kidney function, electrolytes, coagulation function, thyroid function, myocardial enzyme profile, electrocardiogram, and cardiac ultrasound before each cycle of treatment, and chest and abdomen enhanced CT was performed every two cycles to evaluate the efficacy.

### Outcomes

Demographic and clinical data including age, gender, smoking, Child–Pugh class, BCLC stage, HBV infection, HCV infection, extrahepatic metastasis, lymphatic metastasis, macrovascular invasion, AFP, ALT, AST, TBIL, PLT, WBC, BUN, TNM stage, and tumor size were recorded.

1) Tumor responses were evaluated according to RECIST 1.1 (22) and iRECIST (23), including complete response (CR), partial response (PR), stable disease (SD), and progressive disease (PD) for RECIST 1.1. The iRECIST was the same as RECIST 1.1 evaluation at the time points of CR, PR and SD. However, the iRECIST temporarily regarded the PD assessed by RECIST 1.1 as the confirmed progressive disease (iUPD), and the treatment was continued. The iUPD would be re-evaluated according to the reexamined imaging results within 4–8 weeks to confirm the confirmed progressive disease (iCPD). It was worth noting that, in this evaluation mode, the immune complete response (iCR), immune partial response (iPR), and immune stable disease (iSD) could appear again after iUPD. That was, as long as iCPD was not confirmed, it was necessary to continuously evaluate it. In iRECIST evaluation, 12 patients were not evaluated due to lack of reexamined imaging results.

2) Objective response rate (ORR) = (CR + PR)/(CR + PR + SD + PD) × 100%.

3) Disease control rate (DCR) = (CR + PR + SD)/(CR + PR + SD + PD) × 100%.

4) Progression-free survival (PFS) was defined as the time from the onset of treatment to tumor progression or follow-up loss or death.

5) Overall survival (OS) was defined as the time from the onset of treatment to last follow-up or death.

6) Evaluation criteria for adverse events: Common Terminology Criteria for Adverse Events (CTCAE, version 5.0).

### Follow-up

Patients were followed up through a medical record system, an outpatient system, or telephone communication in this study. The last follow-up time was 31 May 2022.

### Statistical analyses

Continuous data that conformed to a normal distribution were expressed as mean ± standard deviation ( x ± s), and were compared by the one-way ANOVA (three groups or more) or the independent *t*-test (two groups). Continuous data that did not conform to the normal distribution were expressed as median (95% confidence intervals), and were compared by the rank sum test (Kruskal–Wallis test). Categorical data were expressed as cases (percentages) and were compared using the Chi-squared test (when all expected values are greater than 1.0 and at least 20% of the expected values are greater than 5) or Fisher’s exact test (when more than 20% of the expected values are less than 5, or at least 1 of the expected values are less than 1). Survival analyses were executed using the Kaplan–Meier method, and the log-rank was used for comparison between the two groups. Data analyses were done with SPSS 24.0 (IBM, NY, USA), and the figures were plotted by GraphPad Prism 9.0 software. Two-sided *p*-value < 0.05 were considered as statistically significant.

## Results

### Baseline characteristics

We gathered a total of 2,182 patients with follow-up data from October 2020 and October 2021, and selected the patients who underwent partial hepatectomy, excluding patients with other operations, and the remaining 1,644 patients. Then, a total of 816 patients were treated with camrelizumab and its combination with apatinib, lenvatinib, and sorafenib after postoperative adjuvant lenvatinib therapy failure, which was considered as disease progression or recurrence detected for the first time after re-evaluation through imaging studies and tumor marker assessments following lenvatinib treatment. Furthermore, we excluded ineligible patients, such as those who were concomitant with other malignancies or severe comorbidities, those who received other treatment protocols in addition to this study after surgery, and whose efficacy had not been or could not be evaluated, and those who met the listed exclusion criteria; 563 patients remained. Then, owing to incomplete data, lost to follow-up, consent withdrawn, etc., only 456 cases remained.

**Figure 1:**
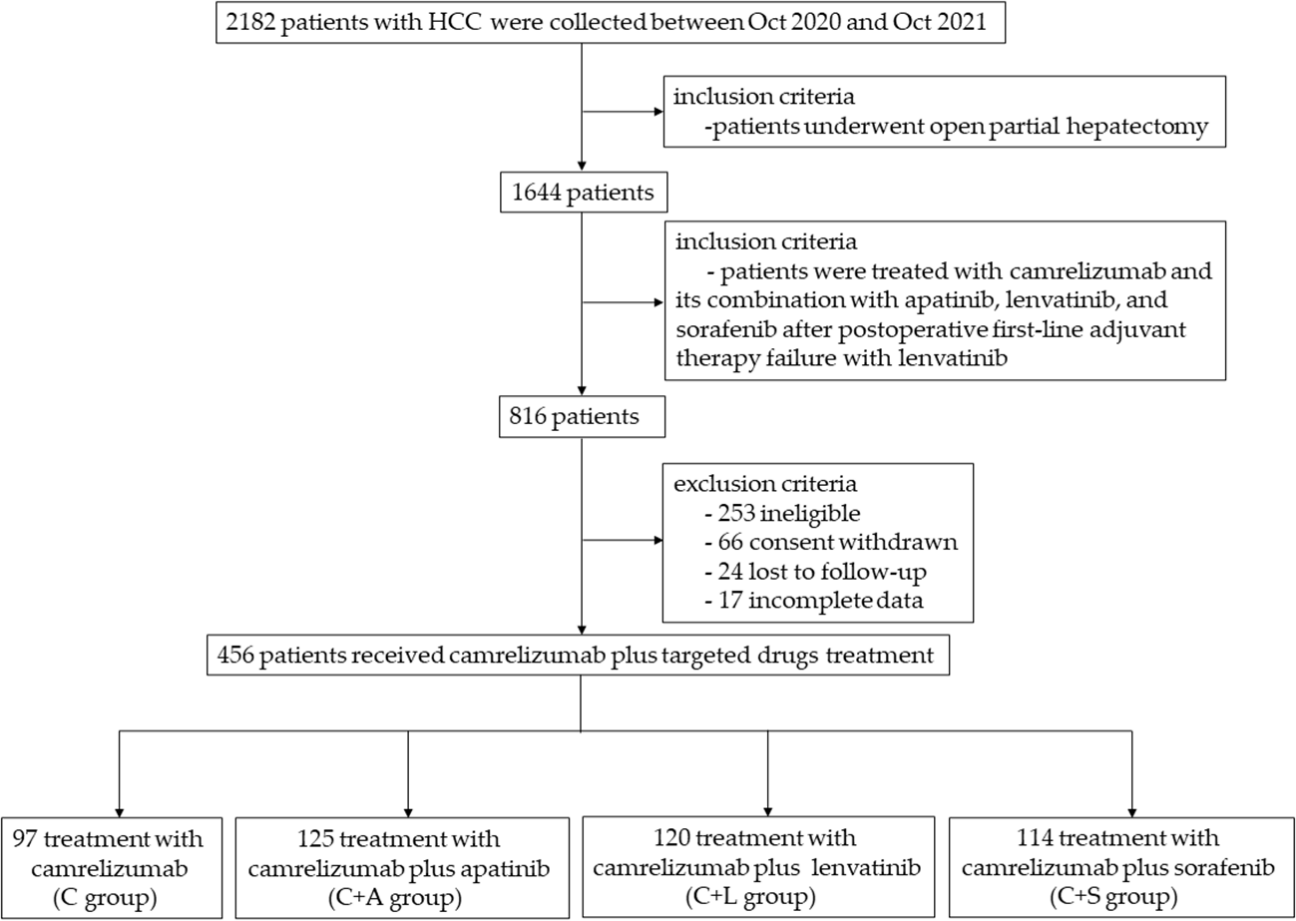
A flowchart for collecting the patients.

Therefore, we collected data from a total of 456 patients with HCC, of whom 97 received camrelizumab monotherapy, 125 patients received camrelizumab plus apatinib therapy, 120 patients received camrelizumab plus lenvatinib therapy, and 114 patients received camrelizumab plus sorafenib therapy (**Figure 1**) during a median follow-up of 13.5 months (IQR, 6.8–16.1). Baseline characteristics of patients are shown in **Table 1**. There were no significant differences in age, gender, smoking, Child–Pugh class, BCLC stage, HBV infection, HCV infection, extrahepatic metastasis, lymphatic metastasis, macrovascular invasion, AFP, ALT, AST, TBIL, PLT, WBC, BUN, TNM stage, and tumor size among the four groups.

**Table 1:** Baseline characteristics.

	C group (*N* = 97)	C+A group (*N* = 125)	C+L group (*N* = 120)	C+S group (*N* = 114)	*χ* ^2^/*F*	*p*
Age, years	51.6 ± 6.3	53.7±4.6	52.4±7.9	52.1±6.6	2.230	0.084
Gender					0.031	0.998
Male	52 (53.6)	66 (52.8)	63 (52.5)	60 (52.6)		
Female	45 (46.4)	59 (47.2)	57 (47.5)	54 (47.4)		
Have a history of smoking	76 (78.4)	101 (80.8)	95 (79.2)	90 (78.9)	0.233	0.972
Child–Pugh class					4.579	0.205
A5	71 (73.2)	103 (82.4)	95 (79.2)	96 (84.2)		
A6	26 (26.8)	22 (17.6)	25 (20.8)	18 (15.8)		
BCLC stage					0.282	0.516
B	22 (22.7)	29 (23.2)	34 (28.3)	23 (20.2)		
C	75 (77.3)	96 (76.8)	86 (71.7)	91 (79.8)		
HBV infection	89 (91.8)	114 (91.2)	104 (86.7)	106 (93.0)	0.3.135	0.371
HCV infection	0	0	0	0	–	–
Extrahepatic metastasis	61 (62.9)	70 (56.0)	76 (63.3)	67 (58.8)	1.797	0.616
Lymphatic metastasis	8 (8.2)	15 (12.0)	10 (8.3)	16 (14.0)	2.851	0.415
Macrovascular invasion	34 (35.1)	40 (32.0)	32 (26.7)	37 (32.5)	1.930	0.587
AFP concentration						
<400 ng/mL	41 (42.3)	72 (57.6)	68 (56.7)	59 (51.8)	6.235	0.101
≥400 ng/mL	56 (57.7)	53 (42.4)	52 (43.3)	55 (48.2)		
ALT (U/L)	48.3 ± 8.6	49.3 ± 11.2	50.4 ± 9.3	49.8 ± 6.4	1.011	0.388
AST (U/L)	59.2 ± 9.7	60.7 ± 8.1	58.8 ± 7.6	61.4 ± 8.9	2.366	0.070
TBIL (μmol/L)	18.7 ± 3.9	19.5 ± 4.5	19.2 ± 3.6	18.3 ± 2.4	2.433	0.064
PLT (10^9^/L)	150.2 ± 11.5	153.8 ± 14.2	149.6 ± 16.1	152.5 ± 13.6	2.303	0.076
WBC (10^9^/L)	6.2 ± 2.1	5.8 ± 1.8	6.4 ± 2.3	6.1 ± 1.4	2.056	0.105
BUN (mmol/L)	4.8 ± 1.2	5.2 ± 1.5	5.0 ± 1.3	5.3 ± 1.8	2.398	0.068
TNM stage						
I/II	74 (76.3)	95 (76.0)	88 (73.3)	85 (74.6)	0.342	0.955
III/IV	23 (23.7)	30 (24.0)	32 (26.7)	29 (25.4)		
Tumor size (cm)	6.6 ± 2.1	7.1 ± 3.3	6.9 ± 1.8	7.4 ± 3.1	1.676	0.658
Preoperative clinical stage					13.445	0.143
I	37 (38.1)	41 (32.8)	48 (40.0)	57 (50.0)		
II	28 (28.9)	45 (36.0)	35 (29.2)	26 (22.8)		
III	22 (22.7)	31 (24.8)	21 (17.5)	20 (17.5)		
IV	10 (10.3)	8 (6.4)	16 (13.3)	11 (9.7)		

BCLC, Barcelona Clinic Liver Cancer; HBV, hepatitis B virus; HCV, hepatitis C virus; AFP, alpha-fetoprotein; ALT, alanine transaminase; AST, aspartate aminotransferase; TBIL, total bilirubin; PLT: platelet count; WBC: white blood cells; BUN, blood urea nitrogen.

### Efficacy

The median follow-up was 13.6 months (95% CI: 11.5–15.3) in the C group, 12.9 months (95% CI: 9.8–15.4) in the C+A group, 14.2 months (95% CI: 12.1–18.5) in the C+L group, and 14.7 months (95% CI: 10.2–17.5) in the C+S group (*p* = 0.342).

Tumor response in the four groups of patients was respectively evaluated by RECIST 1.1 and iRECIST criteria and is shown in **Table 2**.

**Table 2:** Tumor response (RECIST 1.1 and iRECIST).

	C group (*N* = 97)	C+A group (*N* = 125)	C+L group (*N* = 120)	C+S group (*N* = 114)	*χ* ^2^	*p*
	
RECIST 1.1						
CR	5 (5.2)	13 (10.4)	14 (11.7)	9 (7.9)	28.106	0.001
PR	9 (9.3)	39 (31.2)	41 (34.2)	31 (27.2)		
SD	50 (51.5)	45 (36.0)	44 (36.7)	44 (38.6)		
PD	33 (34.0)	28 (22.4)	21 (17.5)	30 (26.3)		
ORR	14 (14.4)	52 (41.6)	55 (45.8)	40 (35.1)	26.495	0.000
DCR	64 (66.0)	97 (77.6)	99 (82.5)	84 (73.7)	8.418	0.038
iRECIST						
CR	5 (5.2)	14 (11.2)	15 (12.5)	10 (8.8)	25.293	0.003
PR	11 (11.3)	40 (32.0)	41 (34.2)	31 (27.2)		
SD	51 (52.6)	45 (36.0)	44 (36.7)	47 (41.2)		
iCPD	26 (26.8)	22 (17.6)	17 (14.2)	25 (21.9)		
Pseudoprogression	3 (3.1)	2 (1.6)	1 (0.8)	4 (3.5)		
Non-evaluable	4 (4.1)	4 (3.2)	3 (2.5)	1 (0.9)		
ORR	16 (17.2)	54 (44.6)	56 (47.9)	41 (36.3)	24.369	0.000
DCR	67 (72.0)	99 (81.8)	100 (85.5)	88 (77.9)	6.352	0.096

Data are expressed as N (%). Intergroup comparisons were made using chi-square tests or Fisher’s exact tests. CR, complete response; PR, partial response; PD, progressive disease; SD, stable disease; ORR, objective response rate; DCR, disease control rate; iCPD, confirmed progressive disease.

For RECIST 1.1 criteria, the ORR in the C group was significantly lower than that of the C+A group (14.4% vs. 41.6%, *χ* ^2^ = 19.295, *p* = 0.000), the C+L group (14.4% vs. 45.8%, *χ* ^2^ = 24.388, *p* = 0.000), and the C+S group (14.4% vs. 35.1%, *χ* ^2^ = 11.741, *p* = 0.001). The DCR in the C group was significantly lower than that of the C+L group (66.0% vs. 82.5%, *χ* ^2^ = 7.832, *p* = 0.005), but not the C+A group (66.0% vs. 77.6%, *χ* ^2^ = 3.701, *p* = 0.054) and the C+S group (66.0% vs. 73.7%, *χ* ^2^ = 1.486, *p* = 0.223). The ORR and DCR in the C+A group and C+L group were higher than that of the C+S group, but the comparison had no statistical difference.

**Figure 2:**
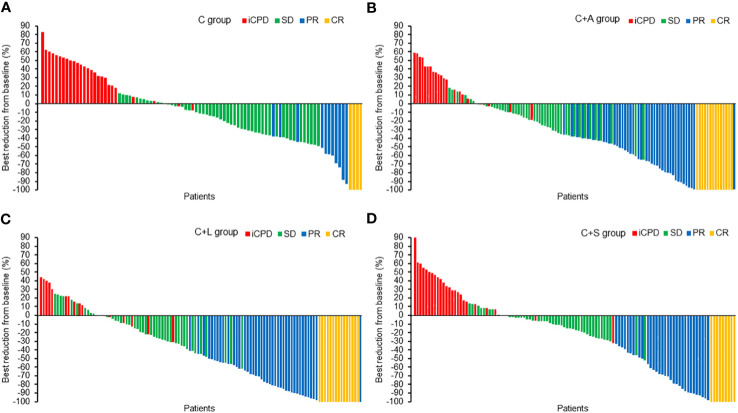
The best reduction from baseline in the longest target lesion. **(A)** C group, **(B)** C+A group, **(C)** C+L group, **(D)** C+S group.

Similarly, for iRECIST criteria, the ORR in the C group was significantly lower than that of the C+A group (17.2% vs. 44.6%, *χ* ^2^ = 17.967, *p* = 0.000), the C+L group (17.2% vs. 47.9%, *χ* ^2^ = 21.617, *p* = 0.000), and the C+S group (17.2% vs. 36.3%, *χ* ^2^ = 9.278, *p* = 0.002). The DCR in the C group was significantly lower than that of the C+L group (72.0% vs. 85.5%, *χ* ^2^ = 5.737, *p* = 0.017), but not the C+A group (72.0% vs. 81.8%, *χ* ^2^ = 2.888, *p* = 0.089) and the C+S group (72.0% vs. 77.9%, *χ* ^2^ = 0.932, *p* = 0.334). Likewise, the ORR and DCR in the C+A group and C+L group were higher than that of the C+S group, but the comparison had no statistical difference. The best changes from baseline in longest target lesion diameter of each patient are shown in **Figure 2**.

Collectively, the C+L group and C+A group exhibited significantly higher ORR and DCR compared to the other two groups.

**Figure 3:**
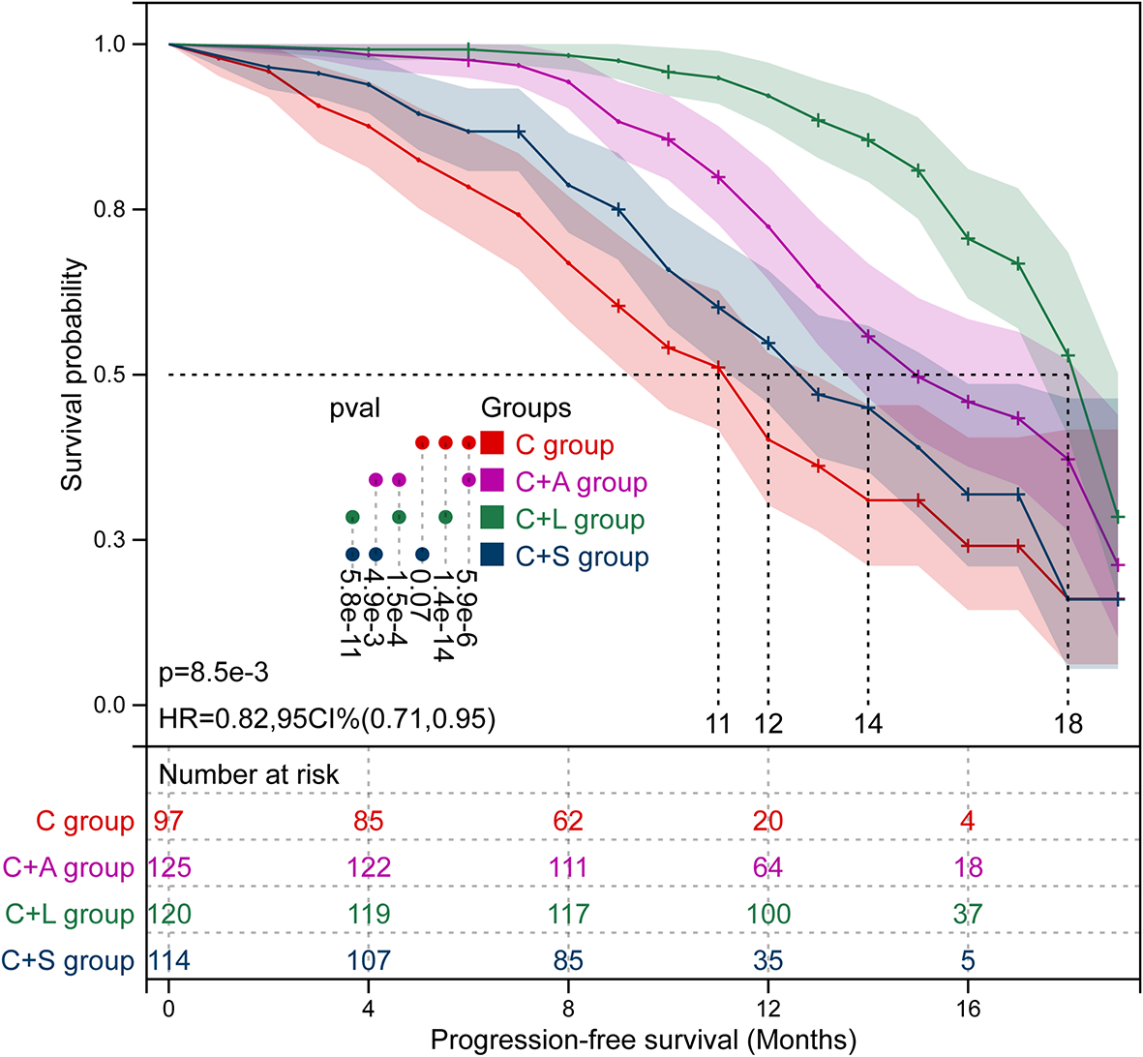
Kaplan–Meier survival curves of PFS in the four groups of patients.

**Figure 4:**
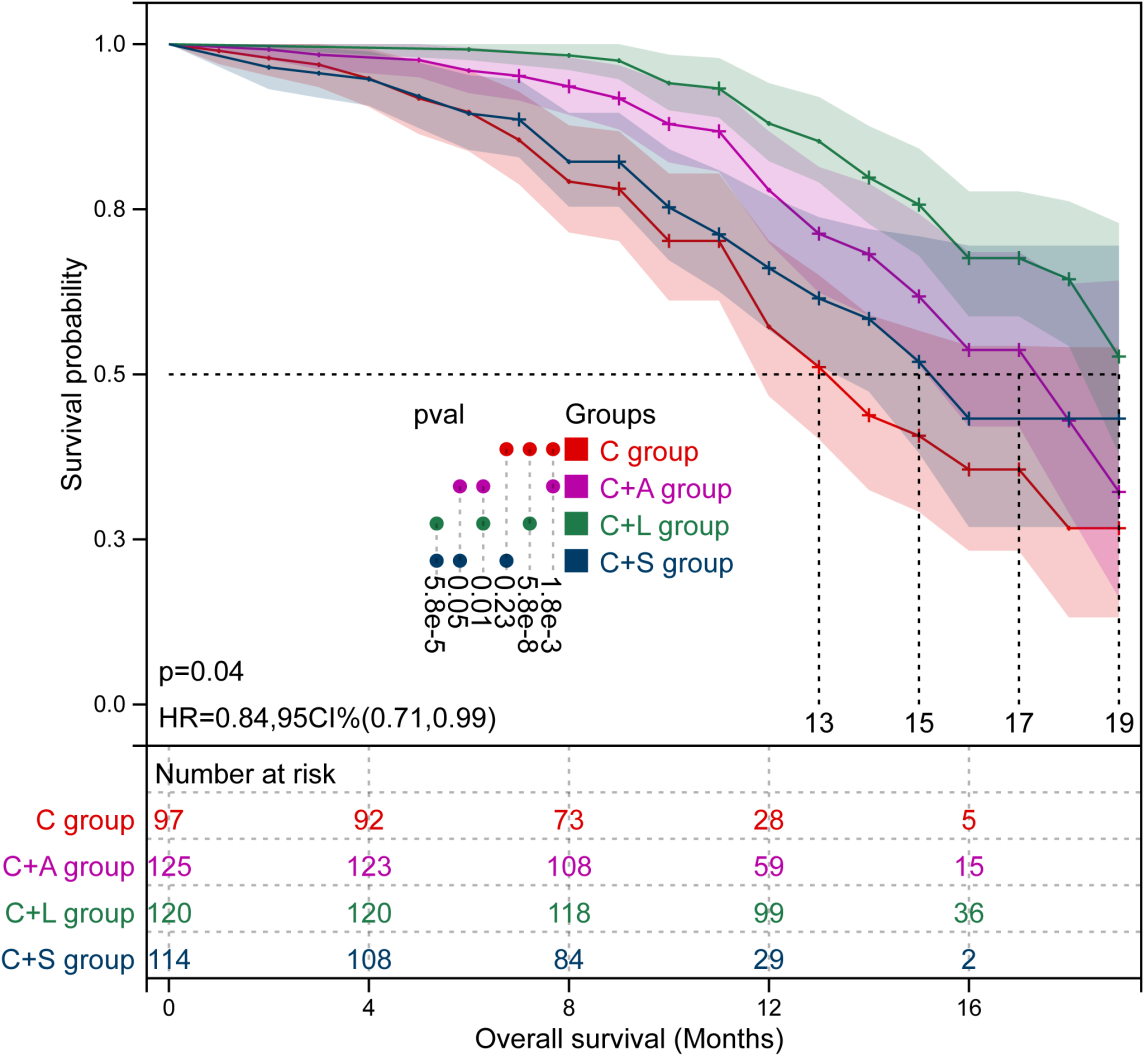
Kaplan–Meier survival curves of OS in the four groups of patients.

Kaplan–Meier survival curves of PFS and OS in the four groups of patients are shown in **Figures 3** and **4**, respectively. The survival data are shown in **Table 3**. The mPFS in the C group was significantly shorter than that of the C+A group (mPFS: 11.0 vs. 14.0, *p* = 5.9e-6) and the C+L group (mPFS: 11.0 vs. 18.0, *p* = 1.4e-14), but not the C+S group (mPFS: 11.0 vs. 12.0, *p* = 0.070). The mPFS in the C+L group was significantly longer than that of the C+A group (mPFS: 18.0 vs. 14.0, *p* = 1.5e-4) and the C+S group (mPFS: 18.0 vs. 12.0, *p* = 5.8e-11). The mPFS in the C+A group was significantly longer than that of the C+S group (mPFS: 14.0 vs. 12.0, *p* = 4.9e-3).

**Table 3:** Survival data.

	C group (*N* = 97)	C+A group (*N* = 125)	C+L group (*N* = 120)	C+S group (*N* = 114)	*χ* ^2^	*p*
mPFS months (95% CI)	11.0 (10.1–12.8)	14.0 (12.7–16.5)	18.0 (16.9–20.1)	12.0 (9.7–14.4)	–	8.5e-3
OS						
6 months, *n* (%)	54 (55.7)	87 (69.6)	84 (70.0)	77 (67.5)	6.321	0.097
12 months, *n* (%)	22 (22.7)	45 (36.0)	78 (65.0)	34 (29.8)	49.338	0.000
Median, months (95% CI)	13.0 (11.6–15.3)	17.0 (15.8–19.4)	19.0 (17.7–20.2)	15.0 (14.1–17.3)	–	0.040

Data are expressed as median (95% CI) and compared by rank sum test (Kruskal–Wallis test), or data are expressed as n (%) and compared by Fisher’s exact test. mPFS, median progression-free survival; OS, overall survival.

The 6-month OS rate in the C group was significantly lower than that of the C+A group (55.7% vs. 69.6%, *χ* ^2^ = 4.573, *p* = 0.032) and C+L group (55.7% vs. 70.0%, *χ* ^2^ = 4.758, *p* = 0.029), but not the C+S group (55.7% vs. 67.5%, *χ* ^2^ = 3.139, *p* = 0.076). The 12-month OS rate in the C group was significantly lower than that of the C+A group (22.7% vs. 36.0%, *χ* ^2^ = 4.598, *p* = 0.032) and C+L group (22.7% vs. 65.0%, *χ* ^2^ = 38.664, *p* = 0.000), but not the C+S group (22.7% vs. 29.8%, *χ* ^2^ = 1.372, *p* = 0.241). The 12-month OS rate in the C+L group was significantly longer than that of the C+A group (65.0% vs. 36.0%, *χ* ^2^ = 20.596, *p* = 0.000) and the C+S group (65.0% vs. 29.8%, *χ* ^2^ = 28.987, *p* = 0.000). The mOS in the C group was significantly shorter than that of the C+A group (mOS: 13.0 vs. 17.0, *p* = 1.8e-3) and the C+L group (mOS: 13.0 vs. 19.0, *p* = 5.8e-8), but not the C+S group (mOS: 13.0 vs. 15.0, *p* = 0.230). The mOS in the C+L group was significantly longer than that of the C+A group (mOS: 19.0 vs. 17.0, *p* = 0.010) and the C+S group (mOS: 19.0 vs. 15.0, *p* = 5.8e-5). Collectively, the C+L group, as well as the C+A group, exhibited significantly prolonged PFS and OS compared to the other two groups.

**Figure 5:**
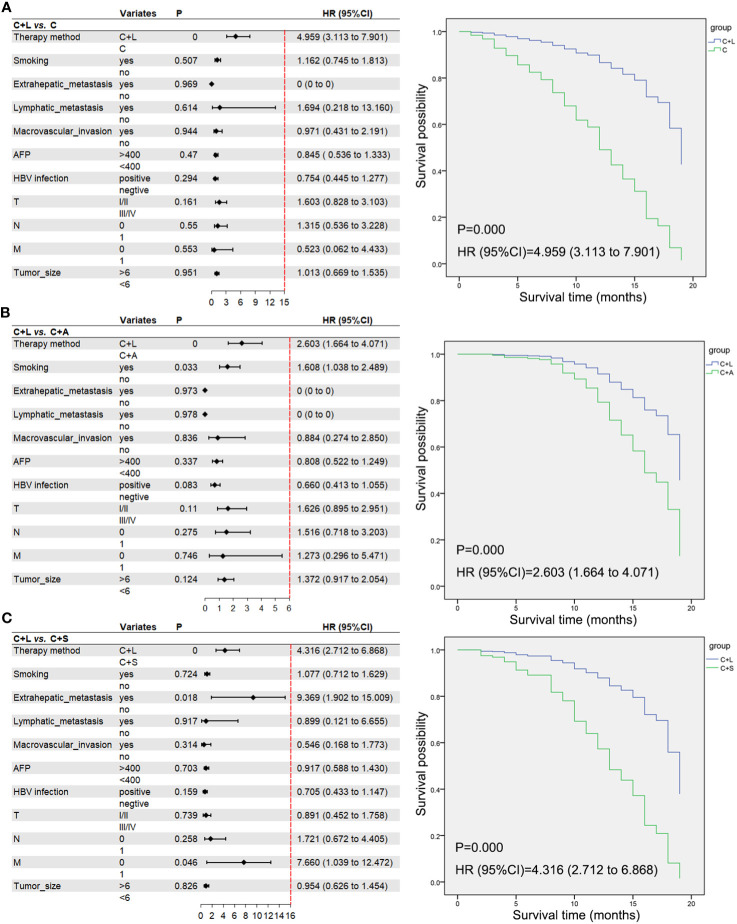
A multivariable Cox regression analysis and the correlative Kaplan–Meier survival curves of OS. **(A)** C+L group vs. C group, **(B)** C+L group vs. C+A group, **(C)** C+L group vs. C+S group. Moreover, we excluded patients with incomplete data and collected pre- and post-treatment AFP .

To account for the potential impact of confounding variables on the prognosis of HCC treatment, we performed a multivariable Cox regression analysis by selecting several factors known to influence HCC prognosis. Additionally, we compared the C+L group as a distinct group to the other three treatment modalities. The analysis revealed that the combination of camrelizumab and lenvatinib demonstrated superior efficacy in the prognosis of HCC compared to the other three treatment modalities (**Figure 5**).

**Figure 6:**
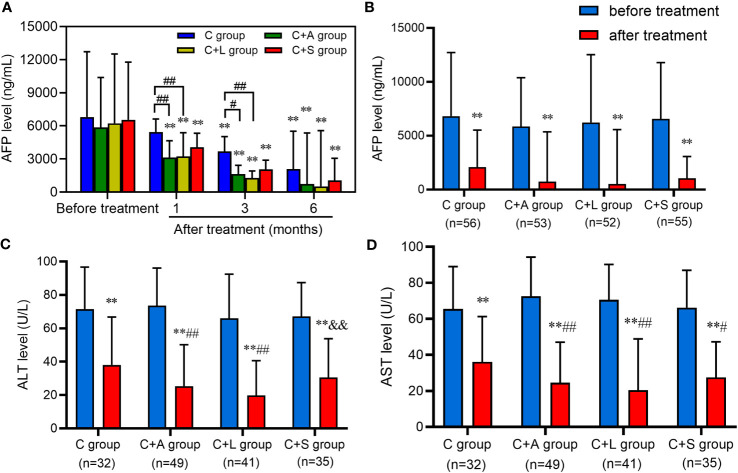
Levels of AFP, ALT, and AST before and after treatment. **(A, B)** AFP, **(C)** ALT, **(D)** AST. ** means *p* < 0.01, compared with before treatment; # and ## respectively mean *p* < 0.05 and *p* < 0.01, compared with the C group after treatment; && means *p* < 0.01, compared with the C+L group after treatment.

Moreover, we excluded patients with incomplete data and collected pre- and post-treatment AFP levels with the first three follow-up examinations. As shown in **Figure 6A**, all treatment groups exhibited a significant reduction in AFP levels compared to baseline. Particularly, both the C+A group and C+L group demonstrated significantly lower AFP levels than the C group at 1 month and 3 months of treatment.

We also collected patients with AFP ≥ 400 ng/mL, and ALT and AST > 40 U/L who were concurrently receiving anti-HBV treatment in each group to analyze the change in the level of these indexes after 6 months of treatment. As shown in **Figures 6B–D**, the AFP, ALT, and AST levels of each group decreased significantly after treatment compared with that before treatment, and the difference was statistically significant (*p* < 0.01). Among them, the AFP level of 6 patients in the C group, 12 patients in the C+A group, 16 patients in the C+L group, and 9 patients in the C+S group recovered to normal levels (AFP < 25 ng/mL). The levels of ALT and AST in the C+A group and C+L group were significantly lower than that in the C group (*p* < 0.01) after treatment. Collectively, patients in the C+A group, especially in the C+L group, had good efficacy compared to the other two groups.

**Figure 7:**
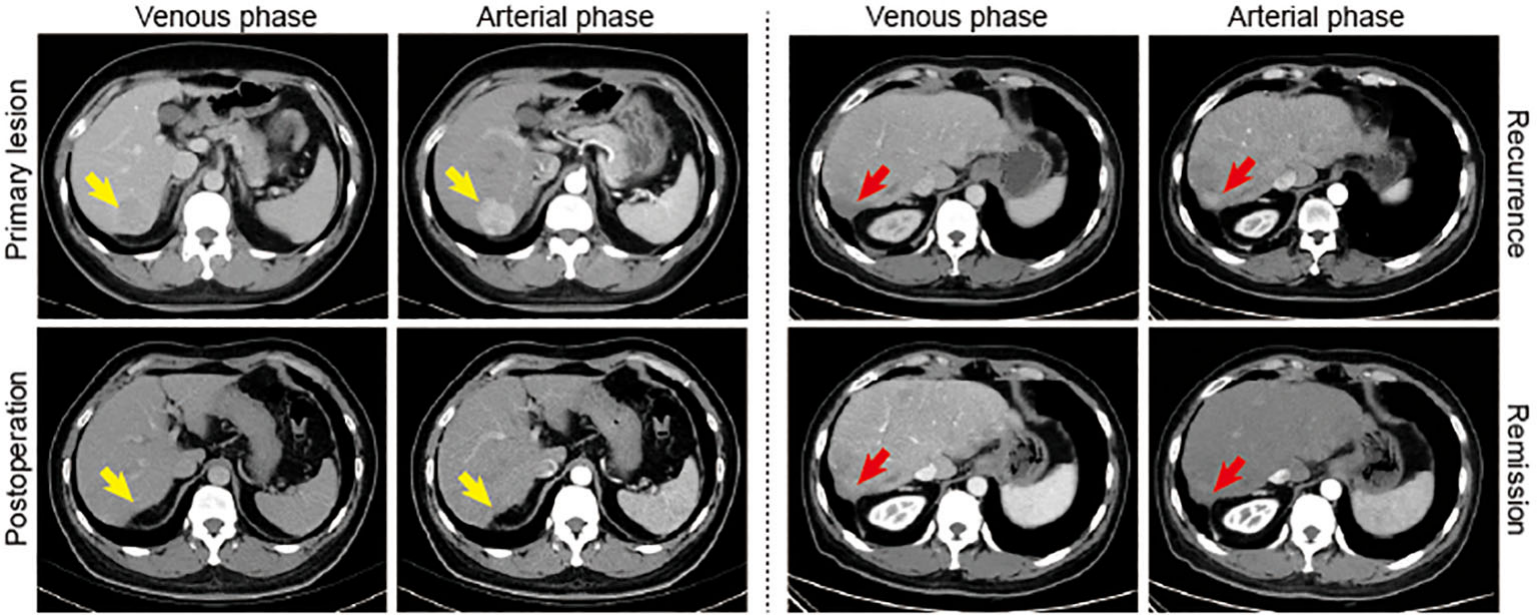
A representative case. Yellow arrows indicate the primary lesion. Red arrows indicate new enhancement lesion in the liver.

The enhanced CT images of a representative patient are shown in **Figure 7**. The patient was initially diagnosed with primary HCC and underwent open partial hepatectomy followed by oral lenvatinib treatment. The first follow-up examination (1 month postoperatively) showed satisfactory recovery. However, during the second follow-up examination (3 months postoperatively), novel enhancement lesions were observed in other locations within the liver. Additionally, serological tests revealed an elevated alpha-fetoprotein (AFP) level compared to the previous examination (579 vs. 162 ng/mL), indicating the possibility of intrahepatic recurrence. Subsequently, targeted immunotherapy (camrelizumab plus lenvatinib) was initiated, leading to significant improvement in the patient’s condition after 6 months of treatment.

### Adverse events

The treatment-related adverse events were evaluated by CTCAE 5.0 and are listed in **Table 4**. The overall incidence of treatment-related adverse events in the C group, C+A group, C+L group, and C+S group was 99.0% (96/97), 96.8% (121/125), 99.2% (119/120), and 98.2% (112/114), respectively. The most common adverse reactions were RCCEP, fatigue, transaminitis, and thrombocytopenia. Although there was no statistical difference in the incidence of adverse events among the four groups, the incidence of adverse events in the C+L group was lower than that in the other three groups. Adverse events with grade 3–4 occurred in 14 patients (14.4%) in the C group, mainly including RCCEP (3 patients, 3.1%), fatigue (10 patients, 10.3%), diarrhea (1 patients, 1.0%), and transaminitis (5 patients, 5.2%), of which 5 patients (5.2%) underwent camrelizumab dose modification on account of adverse events. Adverse events with grade 3–4 occurred in 10 patients (8.0%) in the C+A group, mainly including fatigue (7 patients, 5.6%) and transaminitis (3 patient, 2.4%). Adverse events with grade 3–4 occurred in five patients (4.2%) in the C+L group, mainly including fatigue (four patients, 3.3%) and transaminitis (one patient, 0.8%). Adverse events with grade 3-4 occurred in 11 patients (9.6%) in the C+S group, mainly including RCCEP (3 patients, 2.6%), fatigue (6 patients, 5.3%), and transaminitis (3 patient, 2.6%), of which 2 patients (1.8%) underwent camrelizumab and sorafenib dose modification on account of adverse events. Collectively, the combination of camrelizumab and lenvatinib or apatinib was safe, with no new toxicity signals compared with camrelizumab monotherapy.

**Table 4:** Treatment-related adverse events (CTCAE 5.0).

	C group (*N* = 97)	C+A group (*N* = 125)	C+L group (*N* = 120)	C+S group (*N* = 114)	*χ* ^2^	*p*
Any grade						
Any	96 (99.0)	121 (96.8)	119 (99.2)	112 (98.2)	2.053	0.577
RCCEP	58 (59.8)	73 (58.4)	64 (53.3)	61 (53.5)	1.490	0.685
Fatigue	67 (69.1)	81 (64.8)	71 (59.2)	75 (65.8)	2.463	0.482
Rash	17 (17.5)	23 (18.4)	15 (12.5)	21 (18.4)	2.075	0.557
Pruritus	7 (7.2)	8 (6.4)	2 (1.7)	10 (8.8)	5.906	0.116
Diarrhea	26 (26.8)	22 (17.6)	18 (15.0)	25 (21.9)	5.405	0.144
Nausea	8 (8.2)	13 (10.4)	9 (7.5)	11 (9.6)	0.755	0.860
Headache/dizziness	30 (30.9)	39 (31.2)	31 (25.8)	42 (36.8)	3.307	0.347
Cough	15 (15.5)	8 (6.4)	8 (6.7)	10 (8.8)	6.791	0.079
Decreased appetite	27 (27.8)	29 (23.2)	22 (18.3)	34 (29.8)	4.903	0.179
Immune pneumonia	12 (12.4)	13 (10.4)	6 (5.0)	8 (7.0)	4.634	0.201
Immune cystitis	14 (14.4)	12 (9.6)	10 (8.3)	16 (14.0)	3.185	0.364
Urinary tract infection	5 (5.2)	5 (4.0)	3 (2.5)	6 (5.3)	1.424	0.700
Transaminitis	52 (53.6)	62 (49.6)	45 (37.5)	59 (51.8)	7.290	0.063
Proteinuria	18 (18.6)	13 (10.4)	10 (8.3)	20 (17.5)	7.544	0.056
Hematuria	7 (7.2)	8 (6.4)	4 (3.3)	8 (7.0)	2.032	0.566
Hypothyroidism	5 (5.2)	2 (1.6)	2 (1.7)	4 (3.5)	3.351	0.341
Thrombocytopenia	43 (44.3)	49 (39.2)	36 (30.0)	51 (44.7)	6.795	0.079
Anemia	9 (9.3)	5 (4.0)	3 (2.5)	8 (7.0)	5.806	0.121
Lymphopenia	10 (10.3)	8 (6.4)	6 (5.0)	11 (9.6)	3.075	0.380
Hepatic function abnormal	16 (16.5)	9 (7.2)	8 (6.7)	10 (8.8)	7.527	0.057
Hepatotoxicity	6 (6.2)	5 (4.0)	1 (0.8)	6 (5.3)	4.872	0.181
Grades 3–4						
Any	14 (14.4)	10 (8.0)	5 (4.2)	11 (9.6)	7.268	0.064
RCCEP	3 (3.1)	0	0	3 (2.6)	7.146	0.067
Fatigue	10 (10.3)	7 (5.6)	4 (3.3)	6 (5.3)	4.907	0.179
Rash	0	0	0	0	–	–
Pruritus	0	0	0	0	–	–
Diarrhea	1 (1.0)	0	0	0	3.709	0.295
Nausea	0	0	0	0	–	–
Headache/Dizziness	0	0	0	0	–	–
Cough	0	0	0	0	–	–
Decreased appetite	0	0	0	0	–	–
Immune pneumonia	0	0	0	0	–	–
Immune cystitis	0	0	0	0	–	–
Urinary tract infection	0	0	0	0	–	–
Transaminitis	5 (5.2)	3 (2.4)	1 (0.8)	3 (2.6)	3.950	0.267
Proteinuria	0	0	0	0	–	–
Hematuria	0	0	0	0	–	–
Hypothyroidism	0	0	0	0	–	–
Thrombocytopenia	0	0	0	0	–	–
Anemia	0	0	0	0	–	–
Lymphopenia	0	0	0	0	–	–
Hepatic function abnormal	0	0	0	0	–	–
Hepatotoxicity	0	0	0	0	–	–

RCCEP, reactive cutaneous capillary hyperplasia.

## Discussion

It was difficult for single targeted therapy or single immunotherapy to control tumor progression; either could not increase the survival time and improve the quality of life of patients, resulting in an unsatisfactory efficacy. Studies have confirmed that the ORR of PD-1/PD-L1 inhibitor monotherapy was less than 20% in patients with advanced HCC (24), suggesting that the effect of monotherapy in HCC patients was very limited, and more effective treatment options needed to be explored. In recent years, immunotherapy combined with targeted therapy had become a common treatment method for advanced HCC; compared with single targeted therapy or single immunotherapy, combination therapy had obvious advantages in lesion remission rate, disease control rate, and survival time, as well as in controlling adverse events. Therefore, we sought to explore whether this combination therapy also had a good efficacy on postoperative adjuvant therapy, and which combination had the best curative effect. We retrospectively collected the clinical data of HCC patients treated with camrelizumab monotherapy or camrelizumab plus different targeted drugs (sorafenib, apatinib, and lenvatinib) therapy after postoperative adjuvant lenvatinib therapy failure, and analyzed and compared the clinical efficacy and safety of these treatment protocols to select the optimal one.

PD-1/PD-L1 inhibitor combination targeted therapy had become a research hotspot in the field of HCC system therapy (25). Among them, the phase 1 clinical results of pablizumab combined with lenvatinib in the treatment of advanced HCC showed that the ORR was 42.3% (26). Even more encouraging, on 14 May 2020, the *N Engl J Med* published a phase 3 clinical trial for advanced unresectable liver cancer, which showed that atezolizumab plus bevacizumab reduced the mortality risk by 42% in HCC patients compared with sorafenib (27), making “T+A” the first-line treatment for patients with advanced HCC. In our study of adjuvant therapy, camrelizumab plus different antiangiogenic drug therapies were more beneficial than camrelizumab alone in terms of 12-month ORR, DCR, mPFS, mOS, and OS rates, as well as a safety profile. In clinical work, we found that different PD-1 inhibitors combined with different antiangiogenic drugs had different effects on patients. However, studies comparing the efficacy of different immunotherapies plus different antiangiogenic drug therapies were rare. Therefore, our study further compared the efficacy of camrelizumab plus apatinib or lenvatinib or sorafenib in HCC patients, and found that the 12-month ORR, DCR, mPFS, mOS, and OS rates of camrelizumab plus lenvatinib therapy, as well as camrelizumab plus apatinib therapy, were higher than those of camrelizumab plus sorafenib therapy.

Apatinib, lenvatinib, and sorafenib were all multi-target TKIs whose functions were all anti-angiogenic, but their targets were still different. Apatinib, a selective VEGFR-2 tyrosine kinase inhibitor, could control tumor progression by improving the tumor microenvironment and inhibiting the formation of tumor angiogenesis (28). Hou et al. reported that the overall ORR and DCR of apatinib monotherapy in advanced HCC were 30.4% and 65.2%, respectively, and the mOS and mPFS were 13.8 and 8.7 months, respectively (29). Xu et al. had also reported about camrelizumab combined with apatinib in patients with HCC; the ORR was 34.3% (24/70) in the first line and 22.5% (27/120) in the second line, the mPFS in both cohorts was 5.7 months (5.4–7.4) and 5.5 months (3.7–5.6), and the 12-month OS was 74.7% (62.5–83.5) and 68.2% (59.0–75.7) (30). In our study, the ORR of camrelizumab plus apatinib was 41.6% (52/125) for RECIST 1.1 and 44.6% (54/121) for iRECIST, DCR was 77.6% (97/125) for RECIST 1.1 and 81.8% (99/121) for iRECIST, the mPFS and mOS were 14.0 months (12.7–16.5) and 17.0 months (15.8–19.4), and the 6-month OS and 12-month OS were 69.6% (87/125) and 36.0% (45/125). Briefly, the efficacy of camrelizumab plus apatinib in our study was much higher than that of Xu et al., but the 12-month OS was slightly lower than that of Xu et al., which may be related to the small sample size and short follow-up time of our study.

Lenvatinib could act on VEGFR 1-3, FGFR 1-4, PDGFR α, and other sites, and could effectively control tumor-related changes, including the control of tumor cell reproduction and the inhibition of tumor angiogenesis pathogenicity, which could effectively play an antitumor role (31, 32). The study by Li et al. for camrelizumab plus lenvatinib in unresectable HCC found that the ORR was 37.5% for RECIST 1.1 and 41.7% for mRECIST, mPFS was 10.3 months (6.6–14.0), 12-month OS was 79.2%, and mOS has not been reached (33). A study by Wei et al. on camrelizumab plus lenvatinib as post-progression treatment for advanced HCC indicated that ORR was 28.57%, DCR was 71.43%, and mPFS was 8.0 months (34). In our study, the ORR of camrelizumab plus lenvatinib was 45.8% (55/120) for RECIST 1.1 and 47.9% (56/117) for iRECIST, DCR was 82.5% (99/120) for RECIST 1.1 and 85.5% (100/117) for iRECIST, the mPFS and mOS were 18.0 months (16.9–20.1) and 19.0 months (17.7–20.2), and the 6-month OS and 12-month OS were 70% (84/120) and 65% (78/120), respectively. Collectively, the efficacy of camrelizumab plus lenvatinib in our study was higher than that of Li et al. and Wei et al., the reason might be related to the interaction of lenvatinib with camrelizumab; these viewpoints need to be validated by further studies. Furthermore, consistent with the aforementioned results, the multivariable Cox regression analysis also revealed that the combination of camrelizumab and lenvatinib exhibited a significant advantage in the HCC prognosis compared to the other three treatment modalities.

Sorafenib could not only block tumor angiogenesis by inhibiting VEGFR-2 and PDGFR-β, but also inhibit tumor cell proliferation by blocking the Ras/Raf/MEK/ERK signaling pathway, thereby exerting a dual-inhibition, multi-target blocking anti-HCC effect (35). The study by Liu et al. for advanced HCC patients treated with camrelizumab plus sorafenib showed that the ORR was 17.6% (6/34), DCR was 70.6% (24/34), mOS was 14.0 months (7.2–21.0), and mPFS was 9.5 months (1.2–17.8) (36). In our study, the ORR of camrelizumab plus sorafenib was 35.1% (40/114) for RECIST 1.1 and 36.3% (41/113) for iRECIST, DCR was 73.7% (84/114) for RECIST 1.1 and 77.9% (88/113) for iRECIST, the mPFS and mOS were 12.0 months (9.7–14.4) and 15.0 months (14.1–17.3), and the 6-month OS and 12-month OS were 67.5% (77/114) and 29.8% (34/114), respectively. Taken together, the efficacy of camrelizumab plus sorafenib in our study was basically consistent with the study of Liu et al.

In general, our study showed that camrelizumab plus lenvatinib and camrelizumab plus apatinib appeared to be more effective than camrelizumab plus sorafenib in the adjuvant therapy of HCC patients with the camrelizumab combined with different anti-angiogenic targeted drugs, which was also confirmed by postoperative AFP, ALT, and AST level analysis. The degree of inhibition of FGFR1–4 in lenvatinib exceeded that of sorafenib, which was an important reason why it could observe an improvement in overall efficacy in the field of HCC (17). The reason for the slightly lower efficacy of camrelizumab plus sorafenib in this study might be related to the increasingly common increase in sorafenib resistance (35). Moreover, sorafenib might have a better response to hepatitis C virus (HCV)-infected HCC patients, while Asian HCC patients were mostly infected by hepatitis B virus (HBV) (37).

In terms of safety, a study of camrelizumab plus apatinib in patients with advanced cervical cancer showed that treatment-related adverse events (TRAEs) with grade 3 or 4 occurred in 71.1% of patients; the most common adverse events (AEs) were hypertension (24.4%), anemia (20.0%), and fatigue (15.6%), and the most common immune-related AEs included grade 1–2 hypothyroidism (22.2%) and RCCEP (8.9%) (38). In the study by Xu et al. in HCC patients, the dose of apatinib administered was consistent with our study (250 mg daily), the TRAEs with grade ≥ 3 occurred in 77.4% (147/190) of patients, and the most common AE was hypertension (34.2%) (30). For camrelizumab plus lenvatinib therapy, the most common AE was decreased appetite (41.7%) (33) with some mild or moderate TRAEs (39). Although camrelizumab plus sorafenib therapy exhibited a slightly higher incidence of grade ≥ 3 TRAEs in hand and foot syndrome, diarrhea, transaminitis, and hyperbilirubinemia, most of the TRAEs were controllable (36). In our study, the incidence of TRAEs with grade ≥ 3 had no statistical difference among the four treatment options, but it could still be seen that camrelizumab plus lenvatinib therapy had the least incidence of AEs. Overall, in the treatment of camrelizumab combined with anti-angiogenic targeted drugs, the overall patients could tolerate AEs, but more than 90% of patients still experienced AEs; it was a little comforting that grade 3–4 AEs presented only in 4% of patients for camrelizumab plus lenvatinib therapy. The occurrence of these AEs was closely related to the dose of the drug, and it also caused potential differences in the management of AEs in patients. Among these AEs, RCCEP, fatigue, diarrhea, decreased appetite, transaminitis, and thrombocytopenia all seriously affected the quality of life of patients. Therefore, it was particularly important to promptly and effectively control the AEs that occurred to prevent them from escalating into intolerance and even drug withdrawal.

The combination of PD-1/PD-L1 inhibitors with targeted therapy has emerged as a research hotspot in the field of systemic treatment for HCC. In May 2020, a phase 3 clinical trial published in the *N Engl J Med* reported that the use of atezolizumab in combination with bevacizumab reduced the risk of death by 42% in patients with advanced unresectable HCC compared to sorafenib, establishing the “T+A” regimen as a first-line treatment for advanced HCC. While the “T+A” regimen has become the standard first-line treatment for advanced HCC, it will be crucial in the near future to determine the optimal second-line treatment options and strategies to optimize the selection of the most effective therapies (40). Several completed clinical trials, such as Pembrolizumab+Lenvatinib, Nivolumab+Cabozantinib, and Nivolumab+Lenvatinib, have shown promising results in first-line and second-line treatments for HCC. Ongoing clinical trials, including Regorafenib+Nivolumab, Lenvatinib+Tiselizumab, and Camrelizumab+Lenvatinib as second-line treatment options for HCC, are also eagerly anticipated for their outcomes. It is important to note that Regorafenib, a promising targeted therapy, has demonstrated advantages in both targeted treatment and combination therapy with immunotherapy. Several clinical trials related to Regorafenib are currently underway (41). Thus, while further research is needed to determine the most effective/safe combination of TKIs and ICIs, the synergistic effect of TKIs and ICIs holds great promise. In recent years, besides systemic therapy, significant progress has been made in the field of locoregional treatment for HCC. Transarterial chemoembolization (TACE) has been widely used in the treatment of cancer patients, but its clinical effectiveness remains suboptimal. Recently, a novel technique called super-stable homogeneous iodinated formulation technology (SHIFT) has been developed, revolutionizing the field of catheter-directed arterial chemoembolization (42, 43). This breakthrough in TACE provides another valuable platform for the treatment of HCC.

As a retrospective clinical follow-up study, this study has certain limitations. It is necessary to carry out a multicenter, large sample size and prospective randomized controlled study to confirm the actual efficacy and safety of the therapeutic regimen mentioned in our study, so as to find the drug combination with the greatest benefit. In addition, we also need to improve the follow-up data of our department to study the long-term efficacy of patients.

Overall, the results of this study suggest that camrelizumab plus lenvatinib therapy and camrelizumab plus apatinib therapy have a more satisfactory antitumor activity, a preliminary survival benefit, and a controllable safety profile compared with camrelizumab monotherapy and camrelizumab plus sorafenib therapy. It might be a potential adjuvant therapy or a second-line treatment for patients with HCC.

## Data availability statement

The original contributions presented in the study are included in the article/supplementary material. Further inquiries can be directed to the corresponding authors.

## Ethics statement

This study was approved by the Institutional Ethics Committee of First Affiliated Hospital of Fourth Military Medical University (NO. KY20172013-1). The studies were conducted in accordance with the local legislation and institutional requirements. The participants provided their written informed consent to participate in this study.

## Author contributions

Conceptualization, XL, RD, XW, WC, YQ, H J, and KT; methodology, XW, WC, HJ, JY, WW, FL, and LF; software, XW, HJ, and YQ; validation, JY, WW, FL, and LF; formal analysis, XW, WC, HJ, YQ, JY, WW, FL, and LF; investigation, XW, WC, HJ, YQ, JY, WW, FL, and LF; resources, XL, RD, and KT; data curation, XW, WC, HJ, YQ, JY, WW, FL, and LF; writing-original draft preparation, XW, WC, HJ, and YQ; writing-review and editing, XL, RD, and KT; supervision, XL, and KT; project administration, XL, RD, KT, and XW; All authors contributed to the article and approved the submitted version.
